# Synthesis and pharmacological activities of azo dye derivatives incorporating heterocyclic scaffolds: a review

**DOI:** 10.1039/d2ra04934a

**Published:** 2022-09-13

**Authors:** Kibrom Mezgebe, Endale Mulugeta

**Affiliations:** Department of Applied Chemistry, School of Applied Natural Science, Adama Science and Technology University P.O. Box 1888 Adama Ethiopia endexindex05@gmail.com endale.mulugeta@astu.edu.et

## Abstract

Nowadays, there is significant interest in the synthesis of heterocycle-incorporated azo dye derivatives as potential scaffolds in the pharmaceutical sector. The pharmaceutical or drug industries need a simplistic synthesis approach that can afford a wide range of azo dye derivatives. The incorporation of the heterocyclic moiety into the azo dye scaffold has improved the bioactive properties of the target derivatives. The various biological and pharmacological applications of drugs such as anti-fungal, anti-tuberculosis, anti-viral, anti-inflammatory, anti-cancer, anti-bacterial, DNA binding, and analgesic properties can be easily tuned by introducing heterocyclic moieties. To date, continuous efforts are being made in the search for more potent, new, and safe synthetic methodologies for azo dye derivatives. This review presents a brief discussion of the facile synthetic approaches and the relevance of the title compound and its derivatives towards various biological activities. Thus, the synthesis of azo dye derivatives incorporating heterocyclic scaffolds such as imidazole, pyrazole, thiazole, oxazolone, thiophene, pyrrole, benzothiazole and quinoline moieties and their pharmacological applications are discussed briefly.

## Abstract 2

Nowadays, there is significant interest in the synthesis of heterocycle-incorporated azo dye derivatives as potential scaffolds in the pharmaceutical sector.

## Graphical abstract

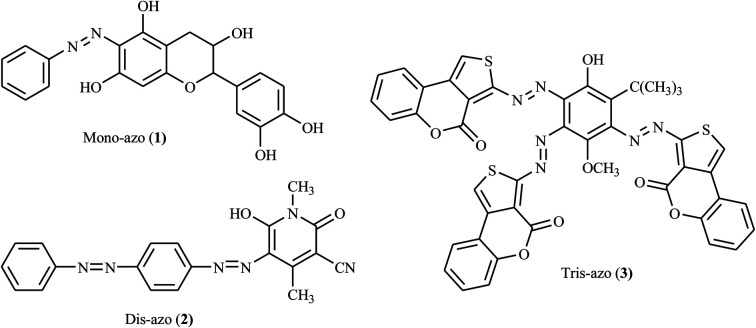

## 1. Introduction

**Fig. 1:**
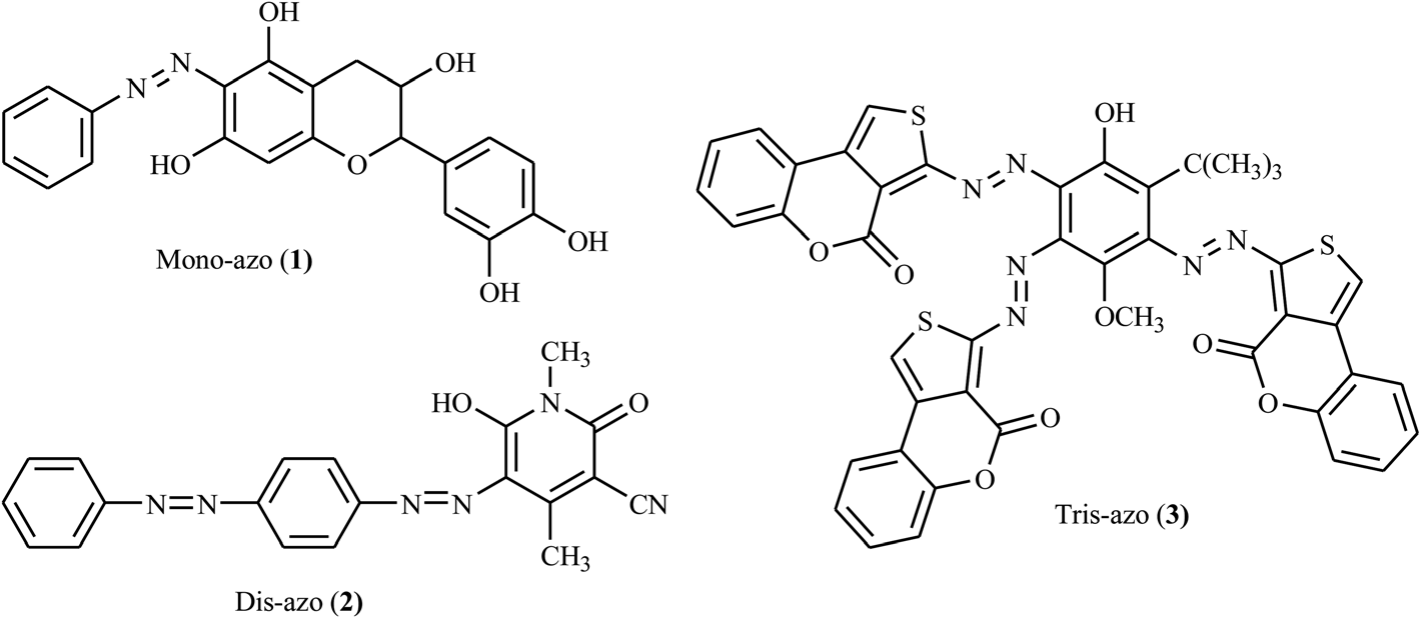
Chemical structures of azo dyes with different numbers of azo groups.

Azo dyes are among the most significant classes of chromophores with diverse applications in the scientific, industrial, and pharmaceutical sectors. Researchers have explored simple and easy synthesis approaches to azo dyes and their derivatives having various potential applications.^1,2^ Azo chromophores are a group of colorant organic materials characterized by the presence of azo groups in the main skeleton structure. There could be two azo groups (dis-azo), for instance, 6-hydroxy-1,4-dimethyl-2-oxo-5-((4-(phenyldiazenyl)phenyl)diazenyl)-1,2-dihydropyridine-3-carbonitrile (2) has two basic azo skeletons, three groups (tris-azo), four groups (tetrakis-azo), or more (poly-azo) in rare cases, see Fig. 1.^3,4^ In addition to their use as colorants in over 50% of all commercial dyes, they have been employed in many applications, such as in inkjet printing, thermal transfer printing, photography, color additives, the biomedical area, molecular recognition, light-controlled polymers, and in the liquid crystal industry.^4^

Azo dyes are generally characterized by their nitrogen–nitrogen double bond (–N=N–) and this structure affords various properties in the textile industries.^4^ In this sense, it is essential for azo dyes to have heterocyclic compounds containing nitrogen, oxygen or sulfur to enhance the color of the dye, leading to different shades with different intensities. Nowadays, azo dyes incorporating heterocyclic moieties exhibit enhanced coloring properties, tinctorial strength, thermal stability, and more positive solvatochromic behavior than the dyes derived from a simple aromatic amine.^5–7^

To date, several synthetic approaches have been developed and reported for the preparation of heterocycle-incorporated azo dyes and their derivatives. The conventional synthesis procedure for the title compound is through diazonium salt coupled with one or more electron-rich nucleophile segments.^8^ In the diazotization procedure, the aromatic or heterocyclic amine is initially converted into a diazonium salt.^9^ The standard reaction of the diazotization reaction occurs at a low temperature in the presence of salts and acid, followed by the resulting diazonium complex interacting with various diazo coupling nucleophile components such as phenol, naphthol, or amine.^10^

**Fig. 2:**
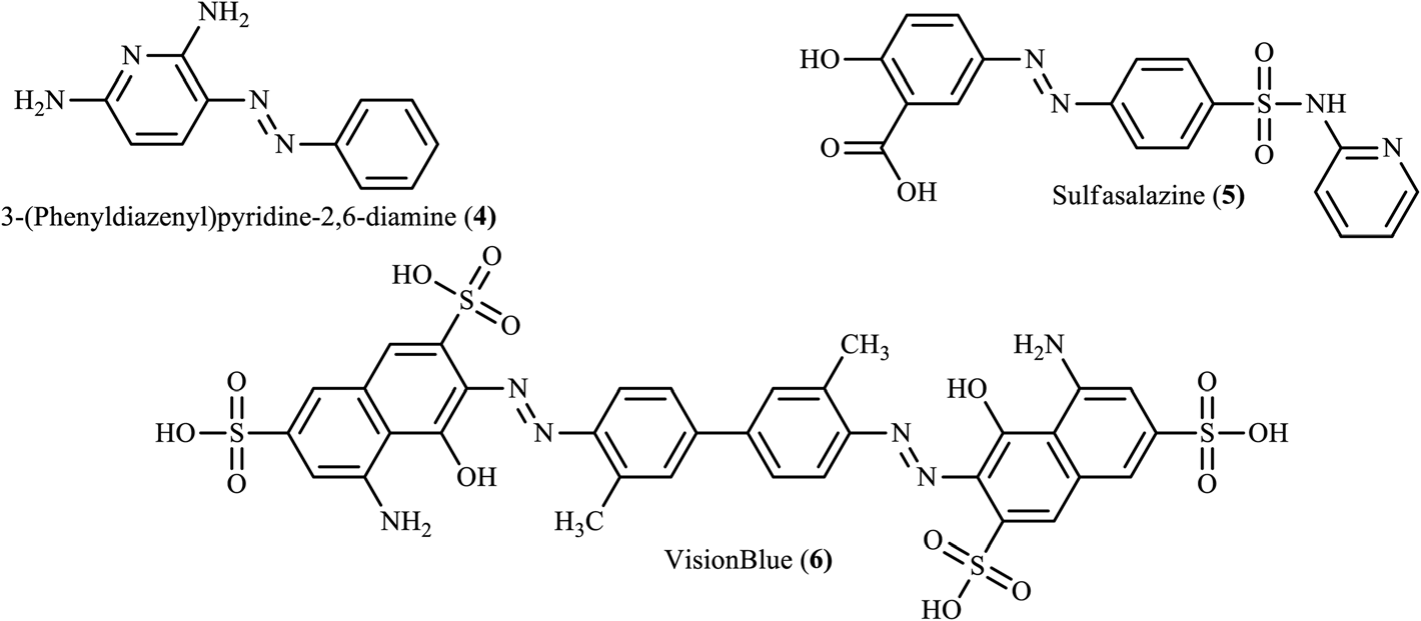
Chemical structures of azo-based drugs.

Although heterocycle-containing azo dye derivatives broadly contribute to pharmaceuticals and drug development, the reports are still not sufficient.^4,11^ Nowadays, the synthesis of heterocycle-containing azo dyes and their derivatives has gained particular attention due to their potent bioactivities such as antimicrobial, antifungal, antiviral, anticonvulsant, antidiabetic, anti-inflammatory, antitubercular, anticancer DNA binding, analgesic properties, and chemosensing activities.^12,13^ Herein, we provide a brief highlight of the synthesis of various heterocycle-containing azo dyes and their derivatives with their potential pharmaceutical activities (Fig. 2).

## 2. Synthesis of heterocycle-containing azo dyes and their derivatives

Nowadays, scholars have given much attention to the suitable design and preparation of the title compounds and their derivatives. So far, various analogs of heterocycle-containing azo dyes and their derivatives have been synthesized and reported *via* different methodologies. This section mainly focuses on the standard and conventional synthesis methodologies of azo dyes containing various heterocyclic moieties.

### 2.1. Azo dyes containing thiophene and its derivatives

**Scheme 1:**
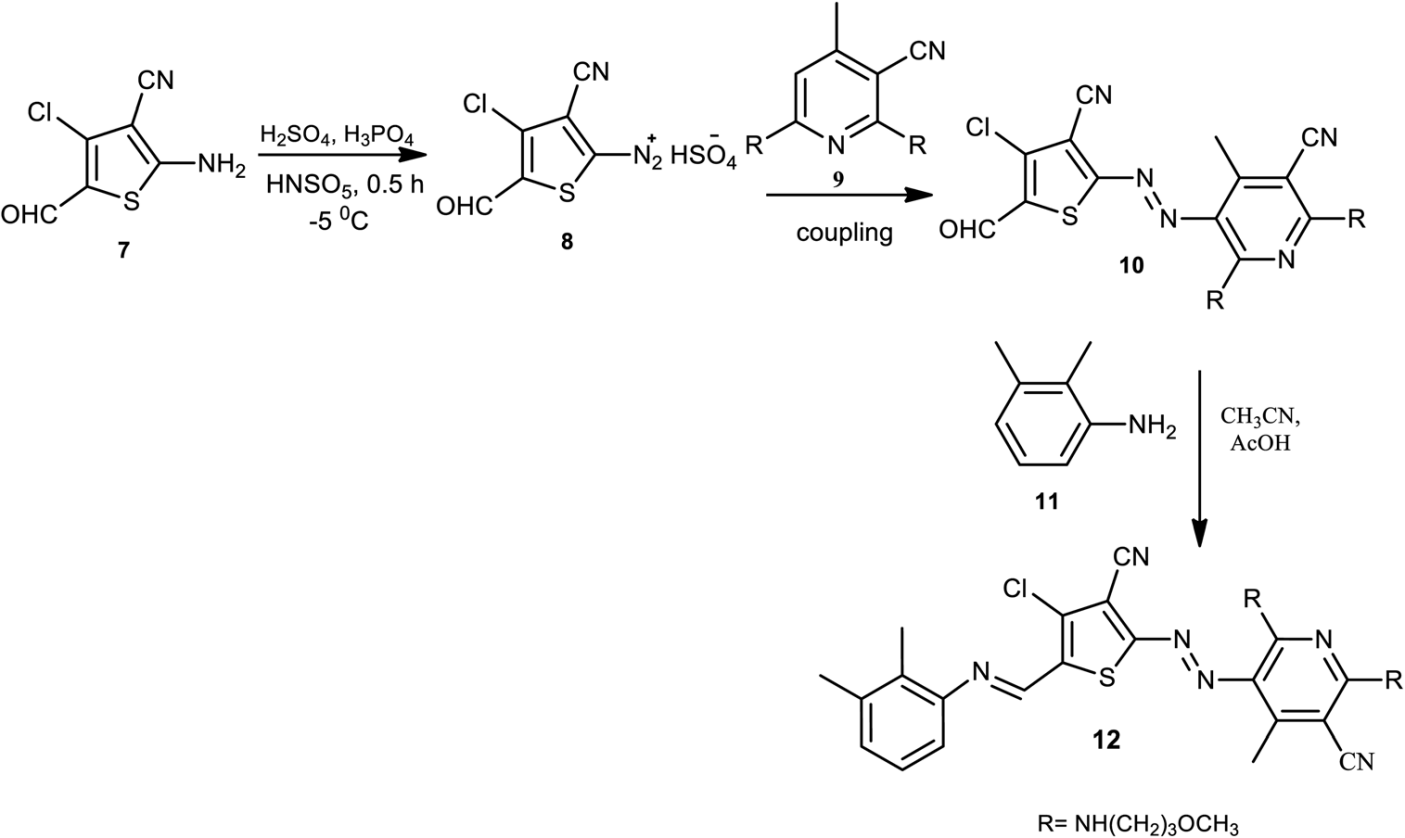
General synthesis route for azo dyes containing thiophene 12.

Wei and coworkers reported the pH-induced azo-keto and azo-enol tautomerism for 6-(3-methoxypropylamino)pyridin-2-one -based thiophene azo dye derivatives.^14^ By linking other functional groups on the azo dye scaffold with the post-modification strategy, the bi-heterocyclic azo dyes 12 were prepared. The diazotization reaction takes place on 3-cyano-4-chloro-5-formylthiophene 7, and 3-methoxypropylamino-substituted pyridine derivatives 9 are used as coupling components to produce 10. The aldehyde of the formylthiophene moiety 10 further reacted with aniline 11 to afford the Schiff base-azo dye 12 with thiophene as a bridge, as described in Scheme 1. Here, the azo dye has a stable pH regardless of the diazo components used since no proton-accepting sites could be found in the pyridine ring; both carbonyl groups are simultaneously replaced by 3-methoxypropan-1-amine.

**Scheme 2:**
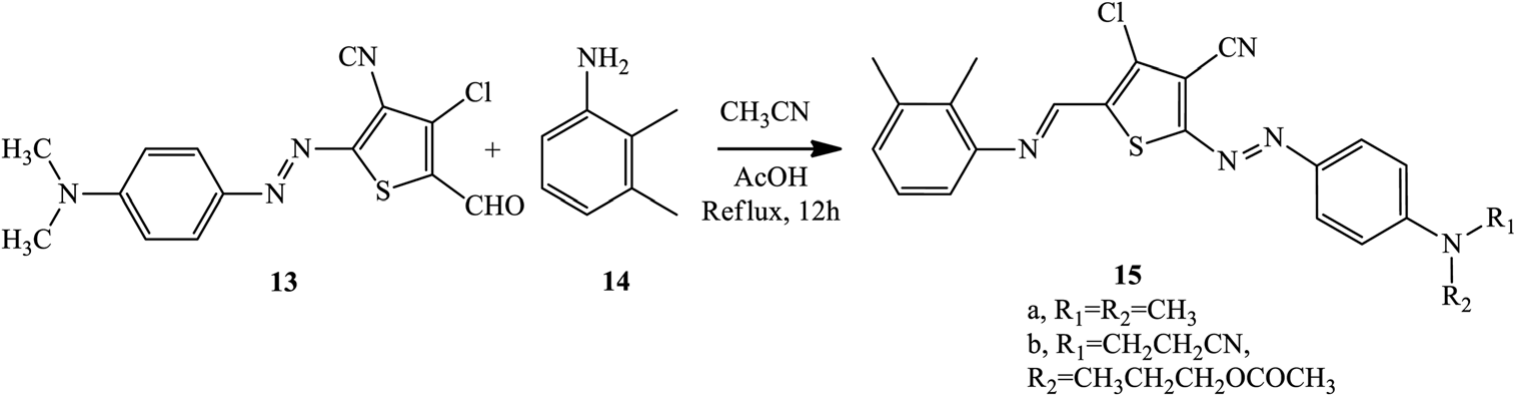
General synthesis route towards Schiff base-azo dye derivative 15.

By modifying the terminal aldehyde radical into an imine version, 2-amino-3-cyano-4-chloro-5 formylthiophene provided the basis for blue-colored heterocyclic azo dyes 15 with an enhanced π-conjugated system, solubility, and electronic spectrum properties of the synthesized compounds.^15^ Azo-azomethine compounds 15 were prepared through a Schiff-base condensation between 2,3-dimethylaniline 14 and the formylthiophene unit of azo dye 13 with various derivatives of aniline-coupling components. The general synthesis route of the dyes is shown in Scheme 2.

### 2.2. Azo dyes containing pyrrole and its derivatives

**Scheme 3:**
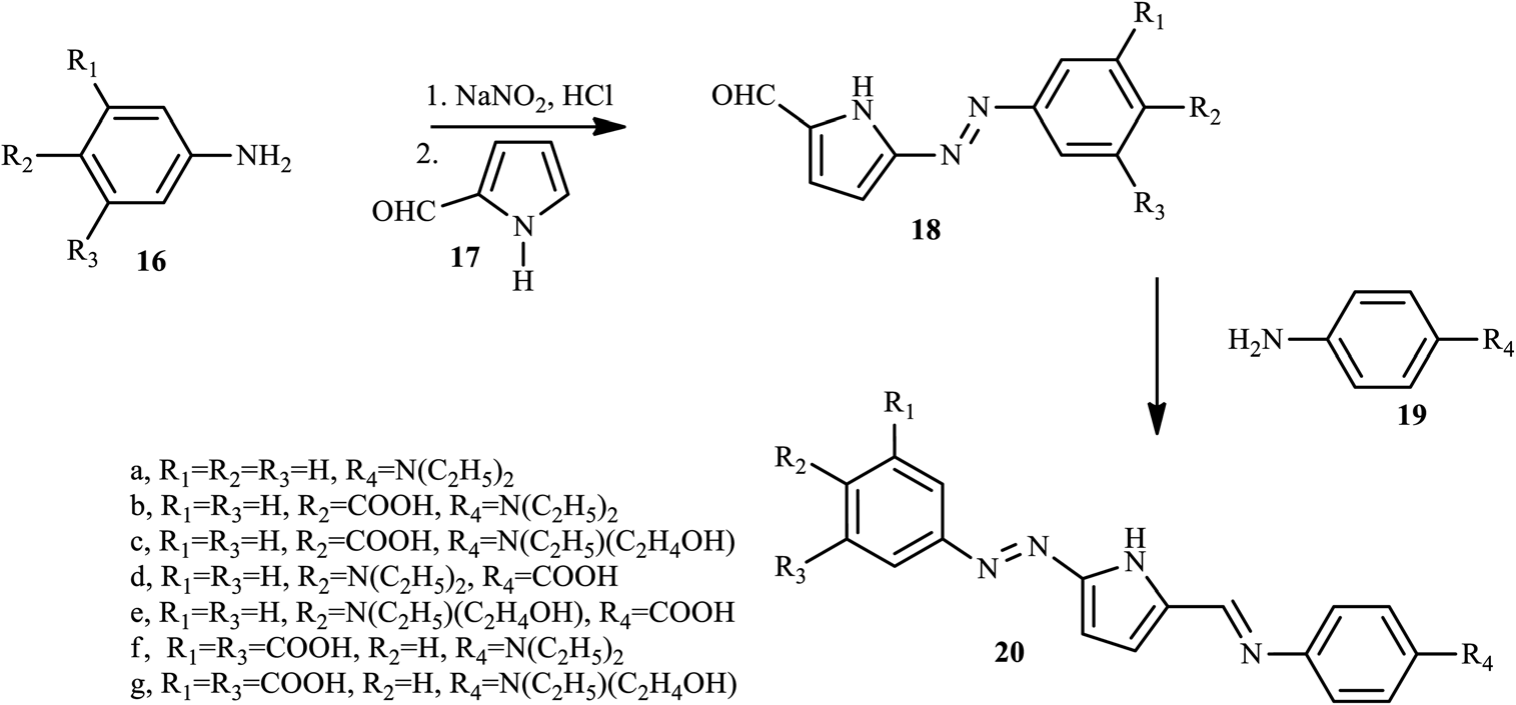
General synthetic route towards azo dye derivatives incorporating pyrrole 20.

Through the diazo coupling reaction scaffold, Maruszewska and Podsiadly synthesized and report novel azo dye pyrrole derivatives 20 containing the azo-1*H*-pyrrole moiety.^16^ During the synthesis of azo dye derivative 20, first aniline, 4-aminobenzoic acid, *N*,*N*-diethyl-*p*-phenylenediamine, *N*-ethyl-*N*-2-hydroxyethyl-*p*-phenylenediamine, and 5-aminoisophthalic acid, respectively, reacted with sodium nitrite/aqueous HCl at 0–5 °C to afford the substituted diazonium salt, and the resulting salt reacted with 1-*H*-pyrrole-2-carbaldehyde 17 in ethanol neutralised with pyridine to produce 18. Finally, compounds 18 condensed with the appropriate aromatic amines 19 in ethanol to give dyes 20 (a–g) as described in Scheme 3. In these dyes, the electron-rich 1*H*-pyrrole moiety was assembled as *p*-bridges in the donor–acceptor *p*-conjugated dye, and aminophenylimine fragments and the carboxyl group were used as donor and anchoring acceptor groups, respectively.

**Scheme 4:**
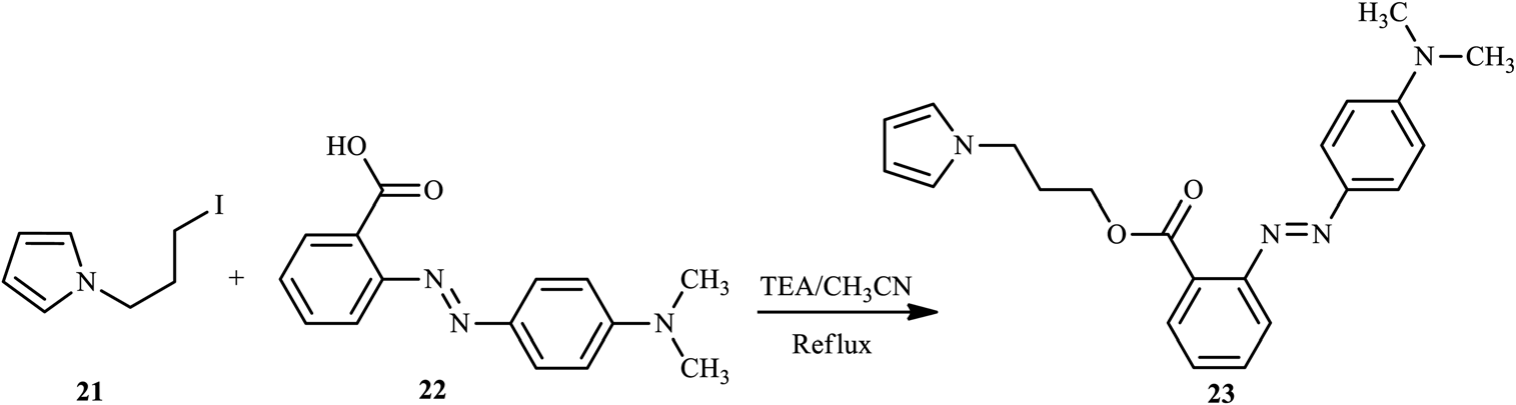
General synthetic route towards azo dye derivatives containing pyrrole 23.

Almeida *et al.* have also synthesized and reported pyrrole azo dye derivative 23 bearing 2-(4-dimethylaminophenylazo) benzoic acid 22, also known as Methyl Red (MR).^17^ The monomer 3-(*N*-pyrrolyl)propyl-2-(4-dimethylaminophenylazo) benzoate (MRPy) 23 was obtained through a simple synthetic route, in mild conditions and with a good yield from 1-(3-iodopropyl)pyrrole and methyl red in the presence of triethylamine, which was added to dry CH_3_CN. The reaction mixture was stirred at 80 °C for 3 h, extracted with H_2_O/CH_3_Cl (1 : 1, v/v), and the crude product was purified after evaporation to give the final product, as shown in Scheme 4. The formation of MRPy was successfully achieved with the addition of boron trifluoride diethyl etherate (BFEE) to electrodes in (C_4_H_9_)_4_NBF_4_/CH_3_CN in the electrolyte system.

### 2.3. Azo dyes containing imidazole and its derivatives

**Scheme 5:**
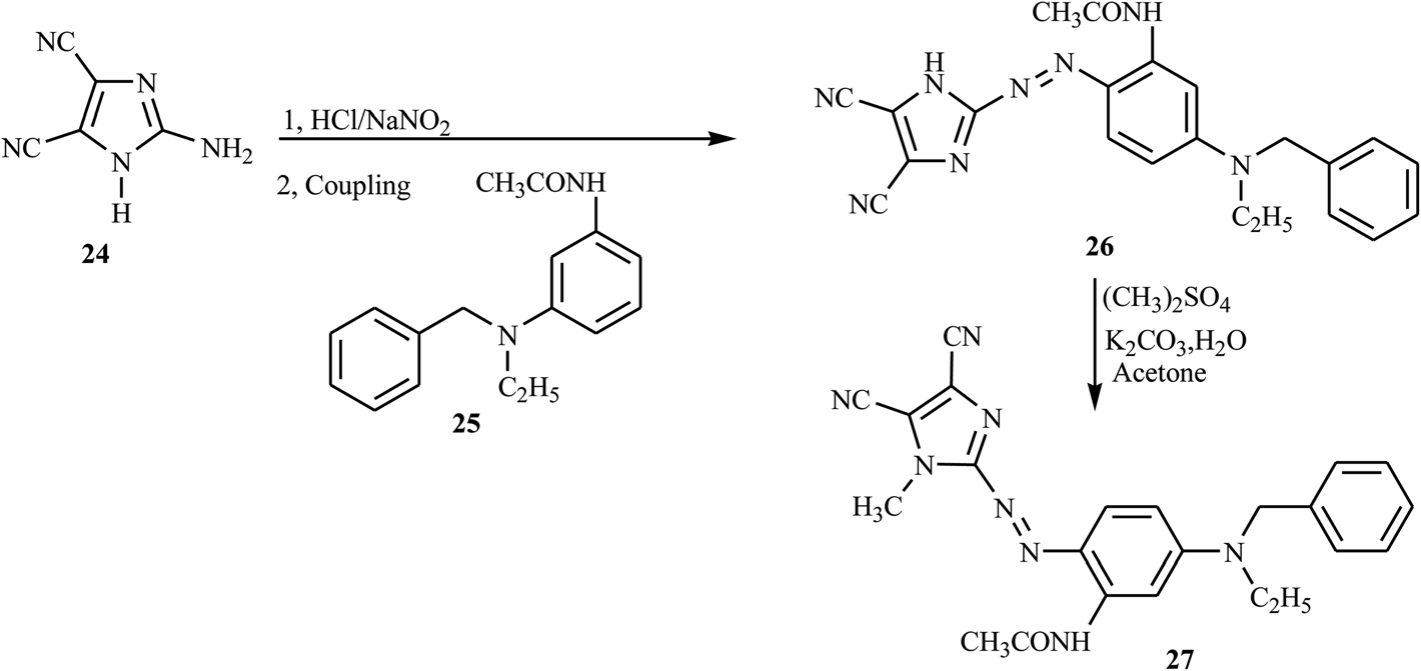
General synthesis route towards azo dye derivatives containing imidazole 27.

New red azo dyes containing imidazole derivative 27 were reported from previous works through the diazo-coupling reaction. The compound 27 was synthesized from the imidazole derivative 24 and passed through the diazotization step in the presence of HCl and NaNO_2_ to obtain the corresponding diazonium salt. The salt was subjected to coupling with *N*-benzyl-*N*-ethyl-*m*-acetamide aniline 25 to afford compound 26 in good yield.^18^ Compound 26 undergoes the methylation reaction through an alkylating agent to methylate the imidazole ring 27 as described in Scheme 5.

**Scheme 6:**
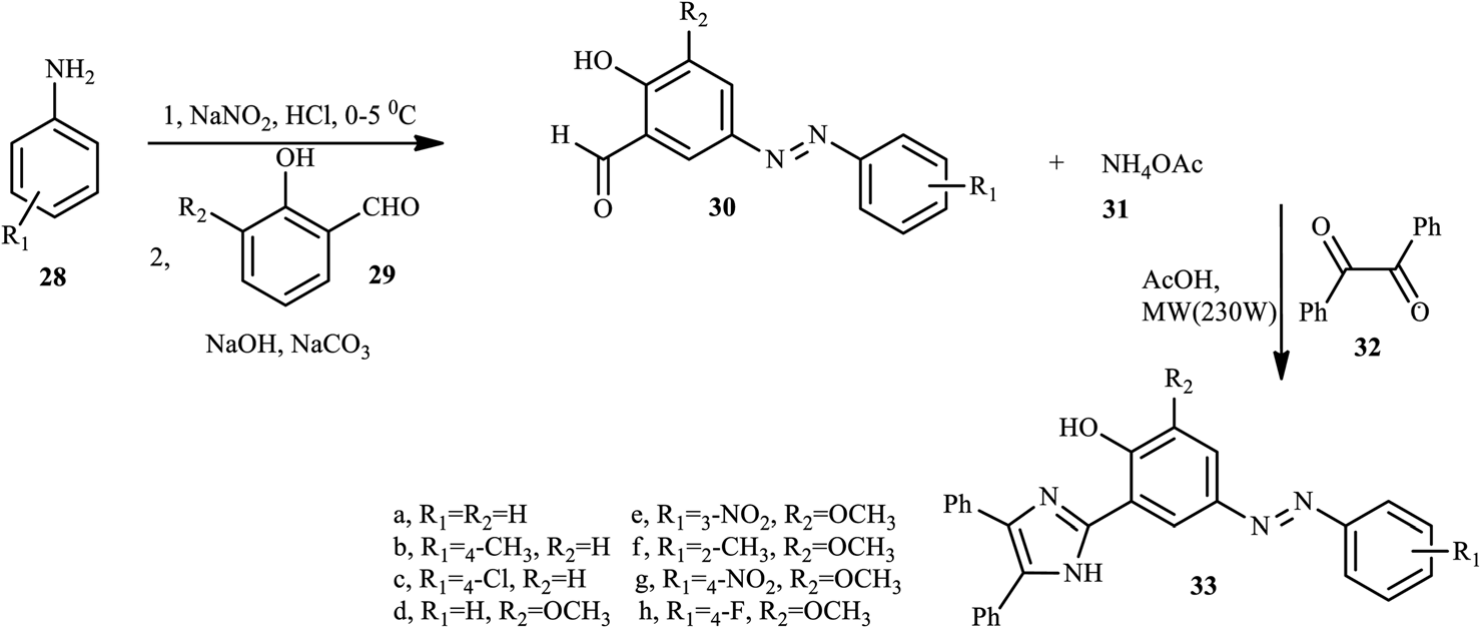
General synthesis route towards azo-imidazole derivatives.

Similarly, through a convenient one-pot three-component synthesis methodology, new azo-imidazole derivatives 33 (a–h) were reported by Mahmoodi *et al.*,^19^ with moderate to excellent yields from the corresponding azo dyes 30, ammonium acetate 31, and benzyl 32 under microwave irradiation in the presence of glacial AcOH as the solvent and organocatalyst in short reaction times. Glacial AcOH is mainly used to activate and enhance the nucleophilic attack of the carbonyl group by ammonia to afford the compound 33. Aniline derivatives 28 were diazotized in the presence of NaNO_2_ and HCl at 0–5 °C and then coupled with the aldehyde derivatives 29 to give the precursor azo dyes 30. The resulting azo dyes 30 were subjected to ammonium acetate 31, followed by a condensation reaction with the benzyl 32, which in turn rearranged to the azo-imidazole 33, as outlined in Scheme 6.

### 2.4. Azo dyes containing pyrazole and its derivatives

**Scheme 7:**
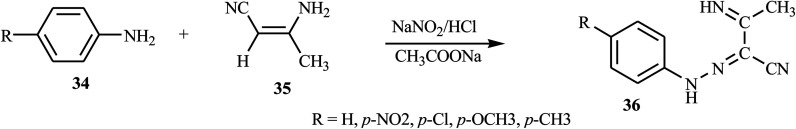
Synthesis of compounds 36.

**Scheme 8:**
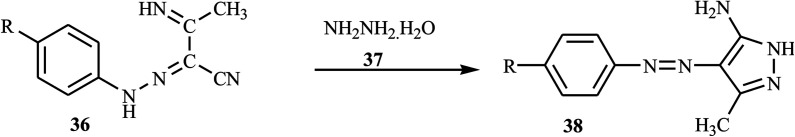
Synthesis of compounds 38.

**Scheme 9:**
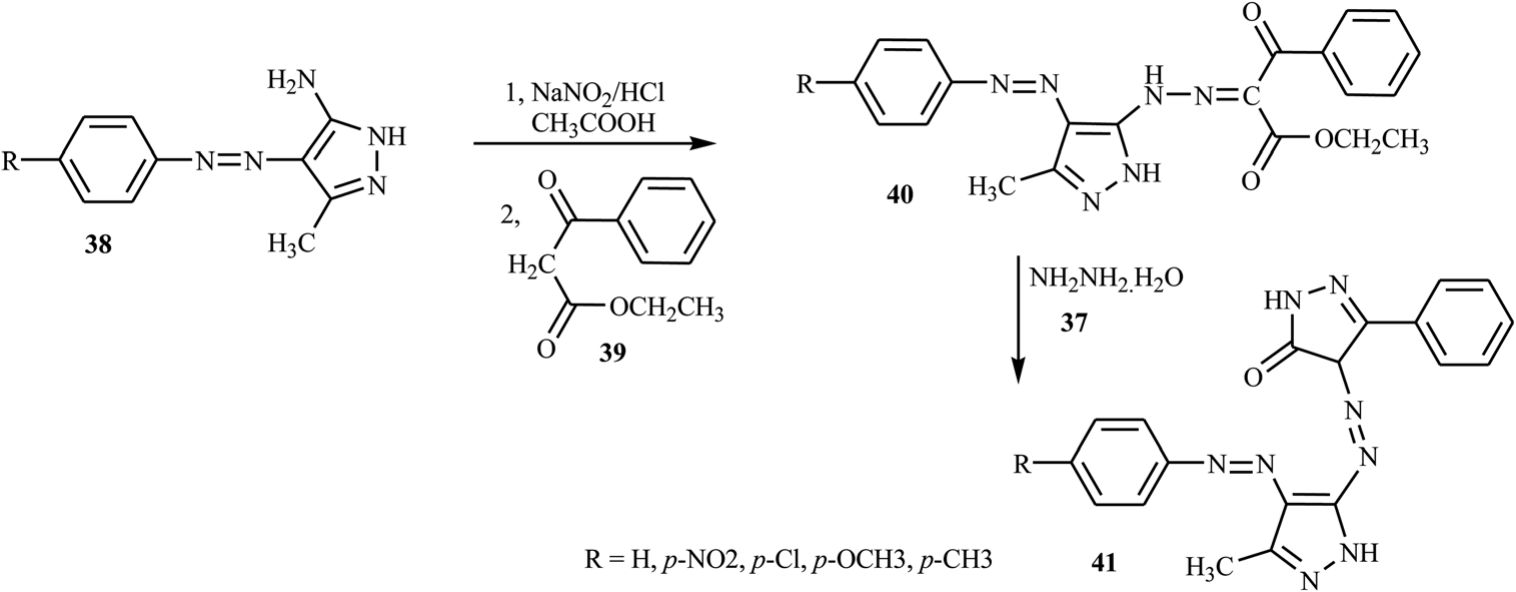
General synthesis route for dis-azo dyes containing pyrazole and pyrazolone 41.

Azo dyes derived from the pyrazole and pyrazolone derivatives have potential broad spectrum biological properties such as anti-bacterial, anti-cancer and antimicrobial activities, and they are used in the pharmaceutical sector.^20^ Demircali *et al.* reported the synthesis of five new azo dyes 41 containing pyrazole derivatives, which were derived from 5-amino-4-arylazo-3-methyl-1*H*-pyrazoles 38, through diazotization followed by a coupling reaction in the presence of hydrazine monohydrate 37; the general route for the synthesis of the dyes is depicted in Scheme 9.^22^ Aniline derivatives 34 were diazotized in the presence of NaNO_2_/HCl followed by coupling with 3-aminocrotononitrile 35 to 2-arylhydrazo-3-ketiminobutyronitriles 36, as outlined in Scheme 7. 2-Arylhydrazo-3-ketiminobutyronitriles 36 were reacted with hydrazine monohydrate 37 to give 5-amino-4-arylazo-3-methyl-1*H*-pyrazoles 38 (Scheme 8). The antibacterial activities of these dyes were evaluated against various pathogenic bacteria and exhibited good-to-excellent activities against the selected strain.^21,22^ The electron-withdrawing groups in the *p*-position resulted in the azo dye becoming more toxic, while the substitution of the electron-donating groups caused the dye to be less toxic.^22^

**Scheme 10:**
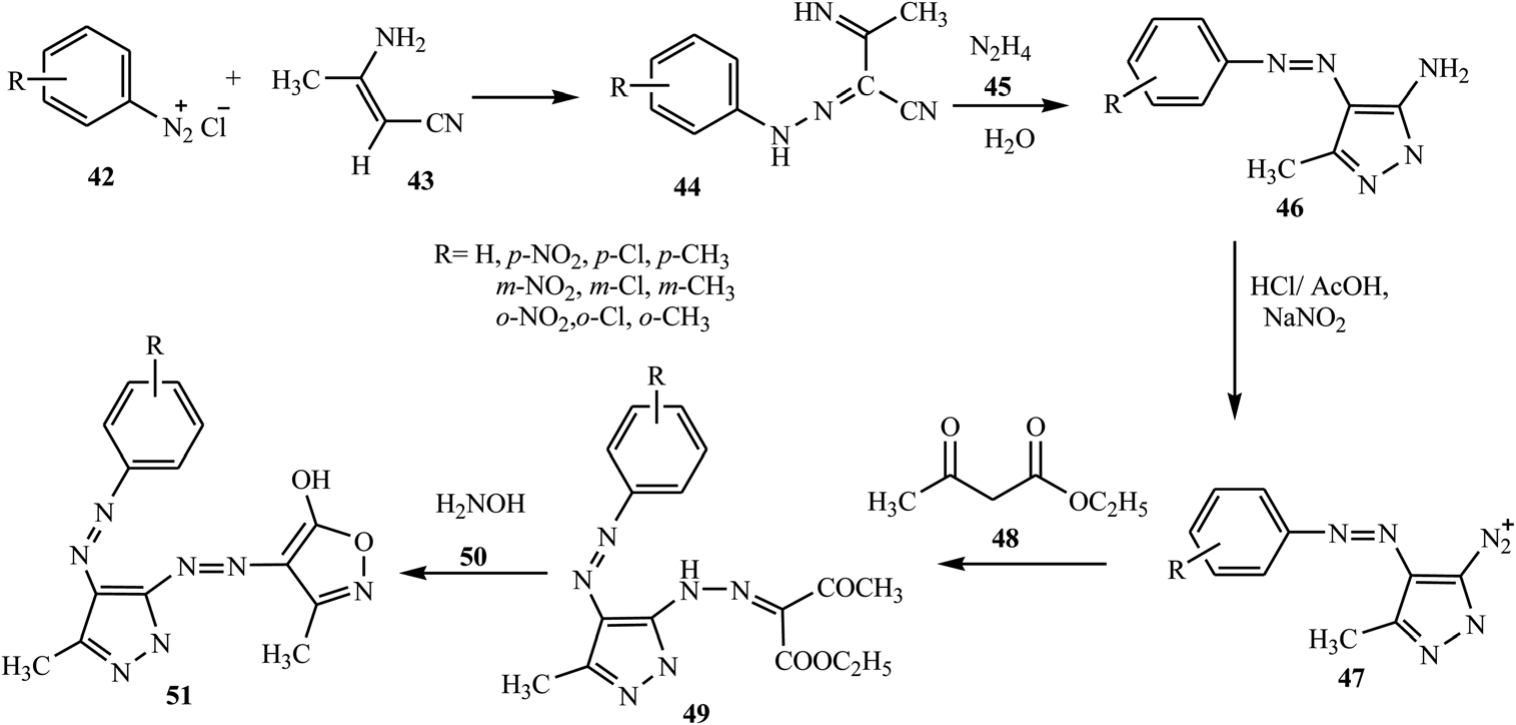
General synthesis route for disazo dyes containing pyrazole 51.

A new series of dispersed disazo dyes 51 containing pyrazole and isoxazole groups were synthesized by a series of synthesis processes.^23^ For these ten newly synthesized disazo-dispersed dyes, one was without auxochrome groups and nine had –NO_2_, –Cl, –CH_3_ auxochromes on *para*, *meta* and *ortho* positions. First, the different aniline derivatives 42 were diazotized and coupled with 3-aminocrotononitrile 43 and coupled to result in the corresponding 2-arylhydrazono-3-ketiminobutyronitriles 44. After the cyclization process of 2-arylhydrazono-3-ketiminobutyronitriles 44 with hydrazine monohydrate 45, 5-amino-4-arylazo-3-methyl-1*H*-pyrazoles 46 were diazotised and coupled with ethyl acetoacetate and produced a series of ethyl pyrazolylhydrazonoacetoacetates 49. The cyclization of 49 with hydroxylamine 50 afforded disazo dyes 51 (Scheme 10).

### 2.5. Azo dyes containing thiazole and its derivatives

**Scheme 11:**
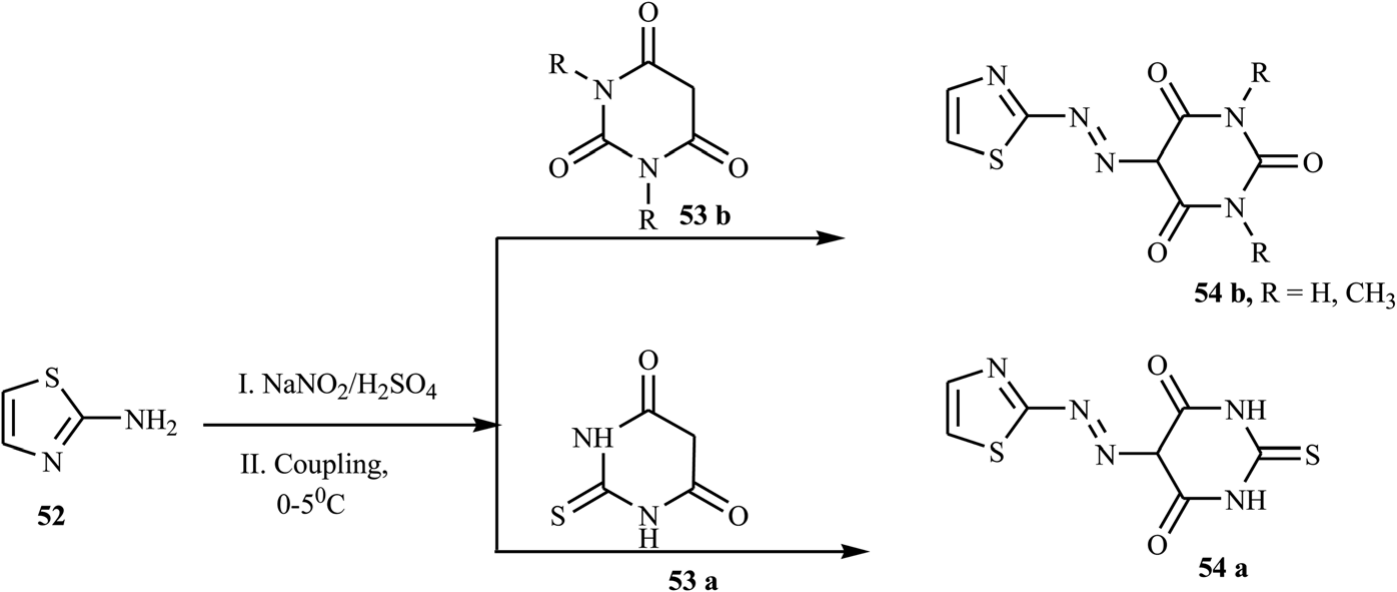
General synthetic route towards azo dye derivatives containing thiazole.

According to the reports, azo dyes containing thiazole have fascinated researchers because of their broad range of pharmacological activities, such as anti-infectious,^24^ antioxidant,^25^ anticancer,^26^ antibacterial, and antifungal.^27^ There have been reports on the synthesis of azo dyes containing the thiazole ring using various methods. Keshavayya *et al.* synthesized three potent anticancer active azo dyes possessing the 2-amino-thiazole moiety *via* a simple, effective, economic, and conventional diazo-coupling reaction.^28^ During the reaction, 1,3-thiazole-2-amine 52 in an acid mixture was reacted with nitrosyl sulphuric acid at 0–5 °C to form the diazonium salt. Azo dye 54 (a & b) was formed when the diazonium salt solution was added to the well-cooled solution of coupling components 53 (a & b) in an aqueous KOH solution. The synthetic route for the preparation of azo dyes is represented in Scheme 11.

**Scheme 12:**
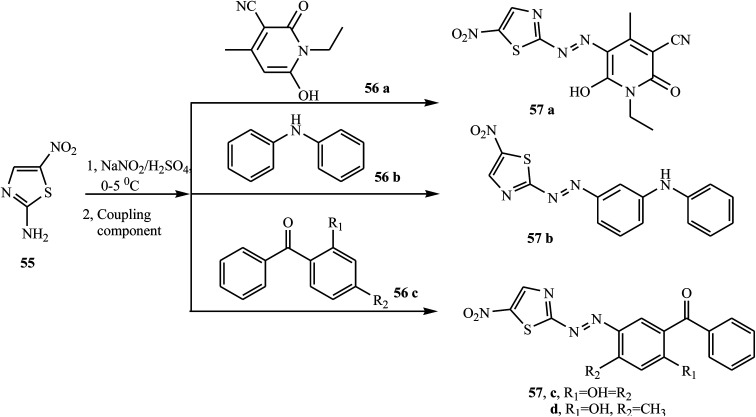
General synthesis route towards azo dye derivatives containing thiazole 57 (a–c).

Similarly, Keshavayya *et al.* reported the synthesis of four new biologically active azo dyes 57 containing thiazole, which were derived from 2-amino-5-nitrothiazole 55 by the conventional diazo-coupling method in an acid condition.^29^ 2-Amino-5-nitrothiazole 55 was diazotized in the presence of sodium nitrite in sulphuric acid and rapidly cooled in an ice bath at 0–5 °C for 10 min. Cold diazonium salt solution was added dropwise with vigorous stirring to the coupling compounds 56 (a–c), which were dissolved in acetic acid, and then the whole reaction mixture was stirred at 0–5 °C for 1 h to give the final azo dyes 57 (a–d), as described in Scheme 12. All the prepared azo dyes revealed promising growth inhibitory effects against selected antibacterial strains and also showed potential antioxidant properties.

### 2.6. Azo dyes containing oxazolone and its derivatives

**Scheme 13:**
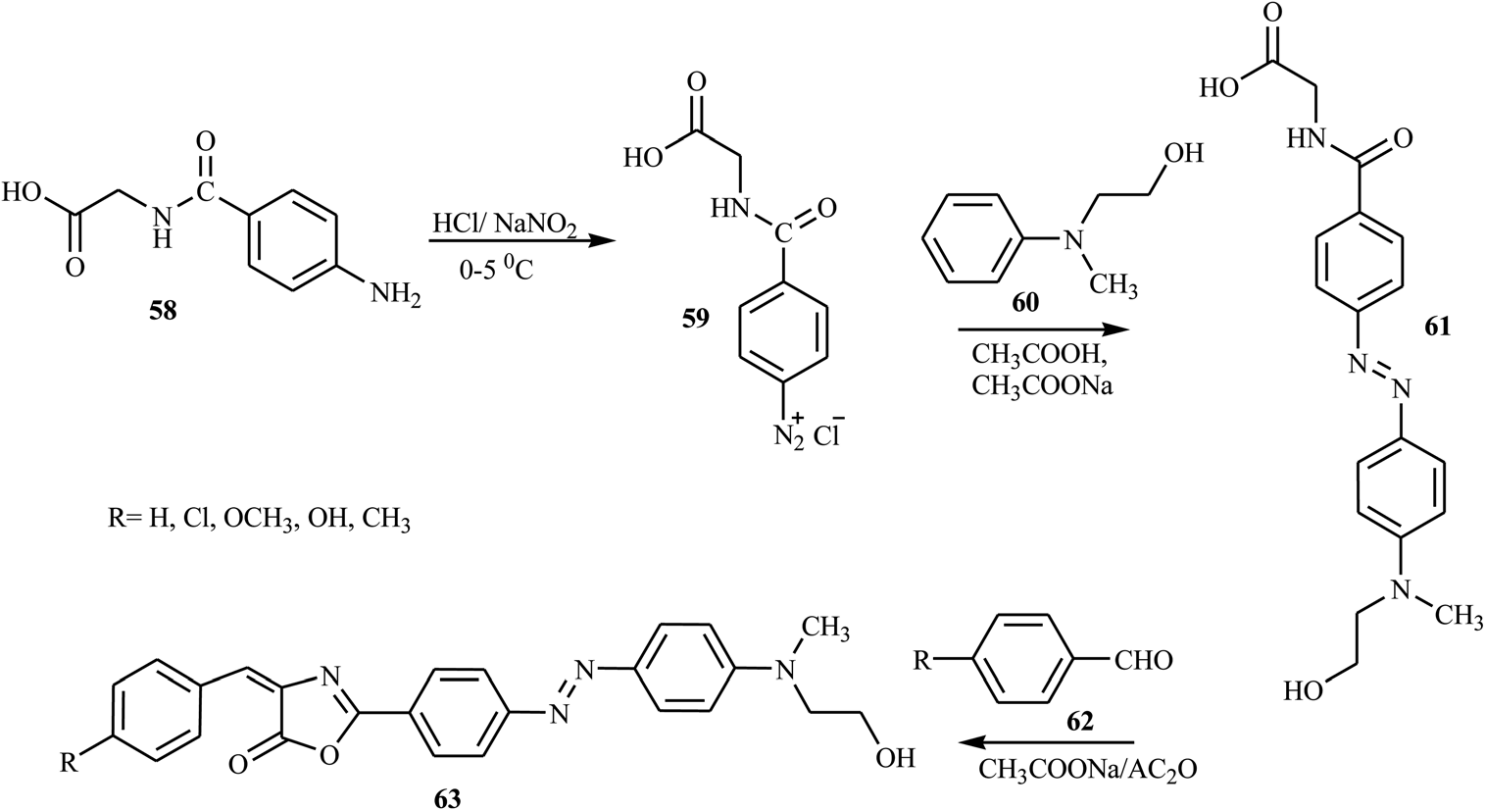
General synthesis route towards azo dye derivatives containing oxazolone 63.

According to Albelwi *et al.*, novel azo dye-containing derivatives of the oxazolone compounds were obtained *via* the Erlenmeyer reaction of the azo dye precursors.^30^ The new 4-arylidene-5-(4*H*)-oxazolone azo chromophore 63 was produced by the condensation of 2-(4-(4-((2-hydroxyethyl)(methyl)amino)phenyl)diazenyl) acetic acid 61 with the corresponding benzaldehydes 62 in the presence of acetic anhydride and sodium acetate, as shown in Scheme 13. Compound 61 was formed from the diazotization of compound 58 followed by the coupling of compound 60. The formation of the unsaturated 5-[4*H*]-oxazolone was elucidated *via* a two-step mechanism. The first step integrates the intermolecular condensation of the azo chromophores 61 in the presence of acetic anhydride to yield the intermediate. This intermediate has two acidic protons that can react with the benzaldehyde derivative 62 in the presence of sodium acetate under refluxing conditions to produce the oxazolone azo dyes 63 in good yields. The synthesized oxazolone-based azo chromophores exhibited strong antifungal and antibacterial activities in comparison to the reference drug *Amphotericin-B*.

**Scheme 14:**
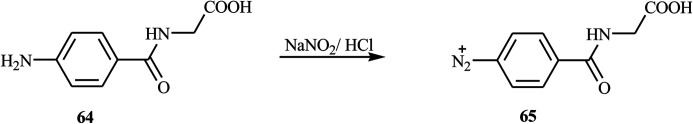
Diazotisation of 4-aminohippuric acid.

**Scheme 15:**
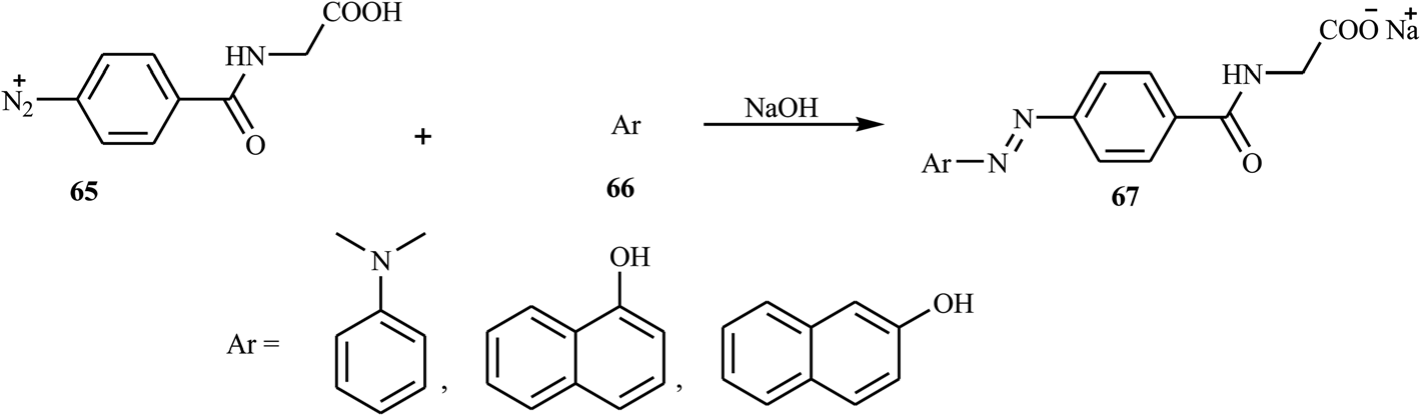
Coupling of the diazonium salt with aromatic compounds.

**Scheme 16:**
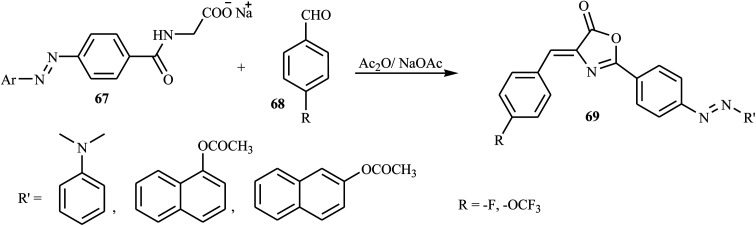
General synthesis route towards azo dye derivatives containing oxazolone 69.

Hamidian and co-workers reported six new potent bioactive azo dyes 69 containing the 5-(4*H*)-oxazolone ring,^31^ by the diazotization of 4-aminohippuric acid 64 and coupling with aromatic derivatives 66 (*N*,*N*-dimethylaniline, 1-naphthol, and 2-naphthol) followed by condensation with benzaldehyde derivatives 68 as described in Schemes 14–16. A mixture of anhydrous sodium acetate, 4-fluorobenzaldehyde or 4-trifluoromethoxy benzaldehyde, sodium salt of azo dye 67 and acetic anhydride was heated with stirring until the mixture was transformed from an orange semi-solid mass into a deep red liquid. After cooling, the precipitated product was filtered and recrystallized in toluene to obtain the final azo dye product 69. All synthesized compounds exhibited high tyrosinase inhibitory behavior.

### 2.7. Azo dyes containing benzothiazole and its derivatives

**Scheme 17:**
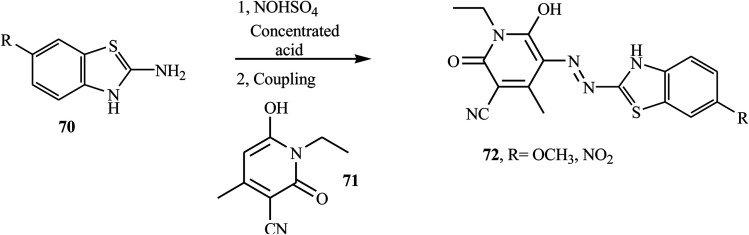
General synthesis route towards azo dye derivatives containing benzothiazole 72.

Song and co-workers reported new bi-heterocyclic dyes 72, which contained *N*-ethyl-3-cyano-4-methyl-6-hydroxy-2-pyridine groups from substituted benzothiazoles.^32^ Under vigorous mechanical stirring at 40 °C, the substituted benzothiazole 70 was mixed with concentrated phosphoric acid, concentrated sulfuric acid, and glacial acetic acid to form the diazonium salt. When the diazonium salt was added dropwise to pyridone derivatives 71 under vigorous mechanical stirring, azo dye 72 was formed. The synthesis route of designed bi-heterocyclic disperse dyes is shown in Scheme 17.

**Scheme 18:**
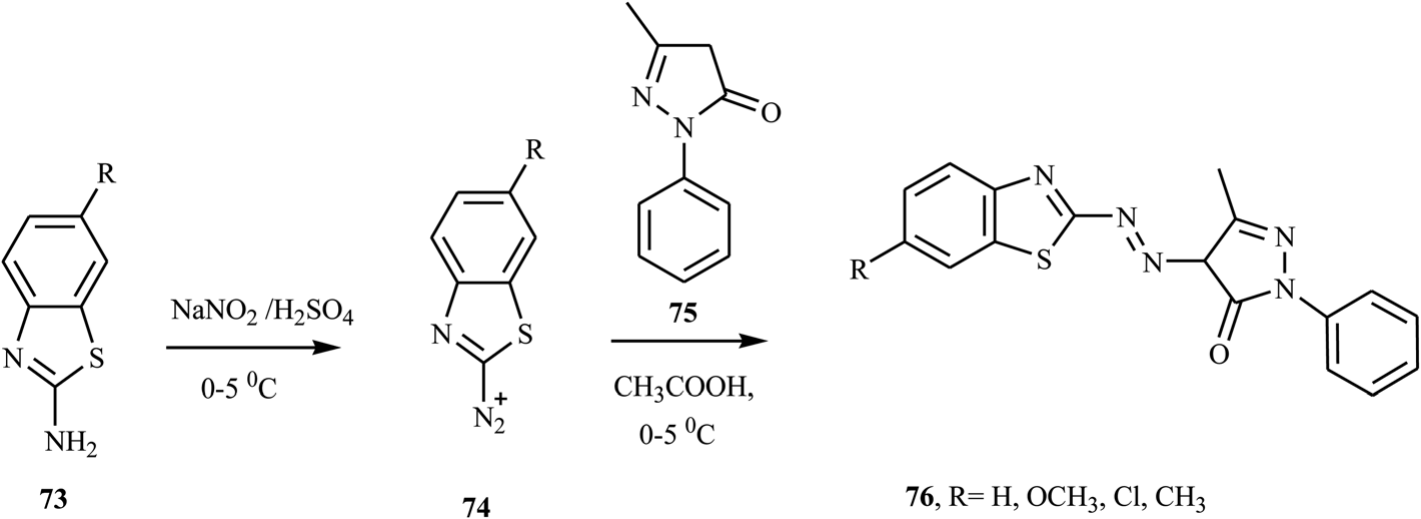
General synthesis route for azo dye derivatives containing benzothiazole 76.

Malayappa and coworkers reported the synthesis of four benzothiazole-based dispersed azo dyes 76 derived from 2-phenyl-2,4-dihydro-3*H*-pyrazole-3-one 75 by diazo coupling electrophilic substitution at 0–5 °C.^33^ 2-Amino-6-substituted benzothiazoles 73 were dissolved in a mixture of glacial acetic acid and propionic acid (2 : 1), and this solution was added dropwise to a well-cooled solution of nitrosylsulfuric acid (NaNO_2_ in H_2_SO_4_) at 0–5 °C to form diazonium salt 74. The resulting diazonium salt 74 coupled with 5-methyl-2-phenyl-2,4-dihydro-3*H*-pyrazol-3-one 75 in acetic acid at 0–5 °C. The precipitated colored product was filtered off, washed several times with distilled water, dried and recrystallized from ethanol. The general route for the synthesis of benzothiazole-azo-pyrazolone dye 76 is outlined in Scheme 18. This compound was described in US patent document 2832761 (1958) for its *application* in the *dyeing* of various textile materials.

### 2.8. Azo dyes containing quinolone and its derivatives

**Scheme 19:**
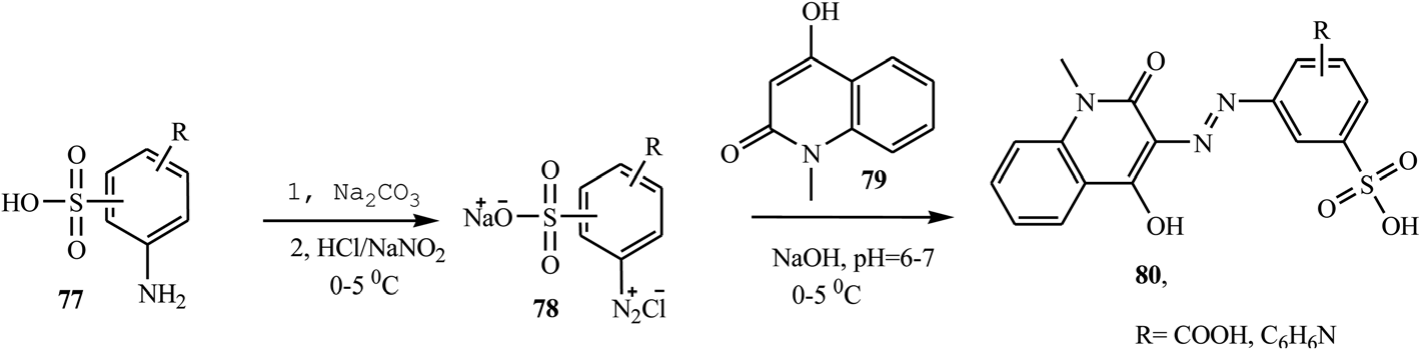
General synthetic route for azo dye derivatives containing quinolone 80.

Shinde and Sekar have reported the synthesis of novel heterocyclic acid dyes 80 by diazotizing various sulphonic acid-based amines and coupling with 4-hyrdoxyl-1-methyl-2-(1*H*)-quinolone.^34^ The dye intermediates, aminosulphonic acids 77 were dissolved in Na_2_CO_3_, cooled to 0–5 °C, and then diazotized in the presence of sodium nitrite and conc. HCl to form 78*via* reverse diazotization and combined with 4-hydroxyl-1-methyl-2-(1*H*)-quinolone 79 to give azo dye 80. A general synthetic route to the preparation of azo dyes is shown in Scheme 19.

**Scheme 20:**
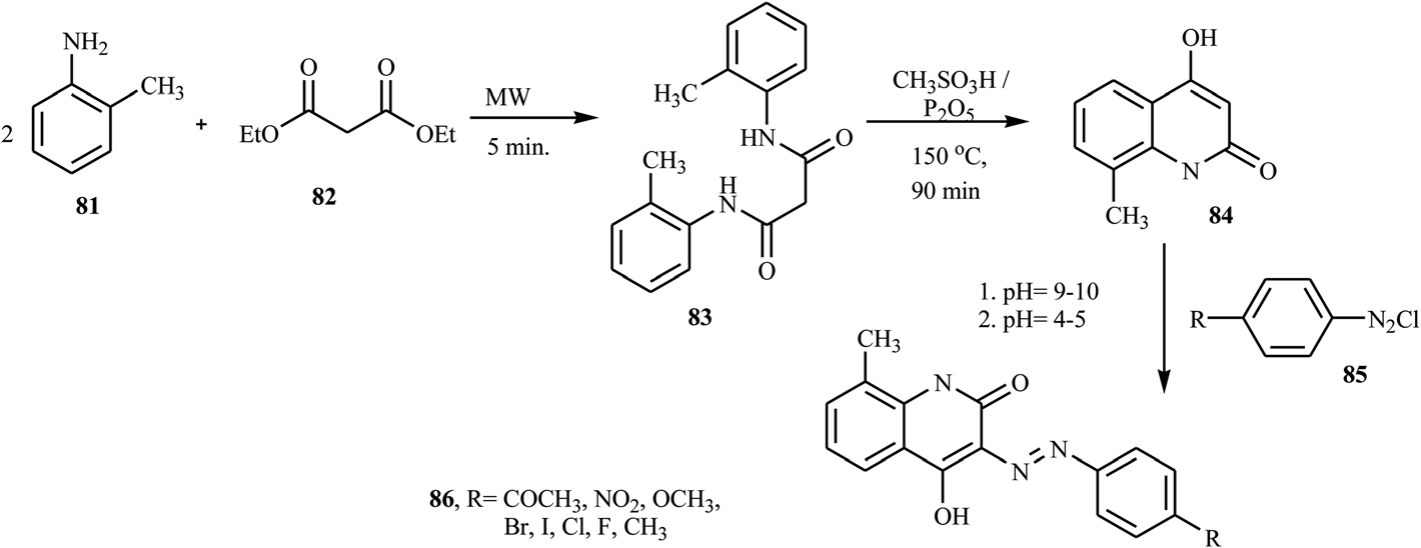
General synthesis route for azo dye-containing quinolone derivative.

Recently, Rufchahi and co-workers reported novel antibacterial-active azo disperse dyes 86, which were synthesized by linking diazotized *p*-substituted aniline derivatives 85 with 8-methyl-4-hydroxyl-2-quinolone 84.^35^ 8-Methyl-4-hydroxyl-2-quinolone 84 was synthesized from the reaction of *N*,*N*′-di-(2-methylphenyl)malonamide 83 with polyphosphoric acid. The malonamide 83 was synthesized by reacting 2-methyl aniline 81 and diethyl malonate 82 in a microwave oven at 320 W for 5 min. The general synthetic routes for the preparation of azo dyes 86 are described in Scheme 20. The dyes exhibited significant antibacterial activity against, *Salmonella enterica*, *Pseudomonas aeruginosa*, *Bacillus subtilis* and *Micrococcus luteus*.

## 3. Biological activity of heterocycle-containing azo dyes and their derivatives

A variety of biological and pharmacological applications were explored for azo dyes that contain heterocycles.^36,37^ Heterocycles are important components of the azo dyes and play an important role in increasing their pharmacological and medicinal properties, such as antibacterial,^36,38^ antioxidant,^39^ anticancer and antitumor,^40^ and anti-inflammatory activities.^37^

### 3.1. Antibacterial activity

**Fig. 3:**
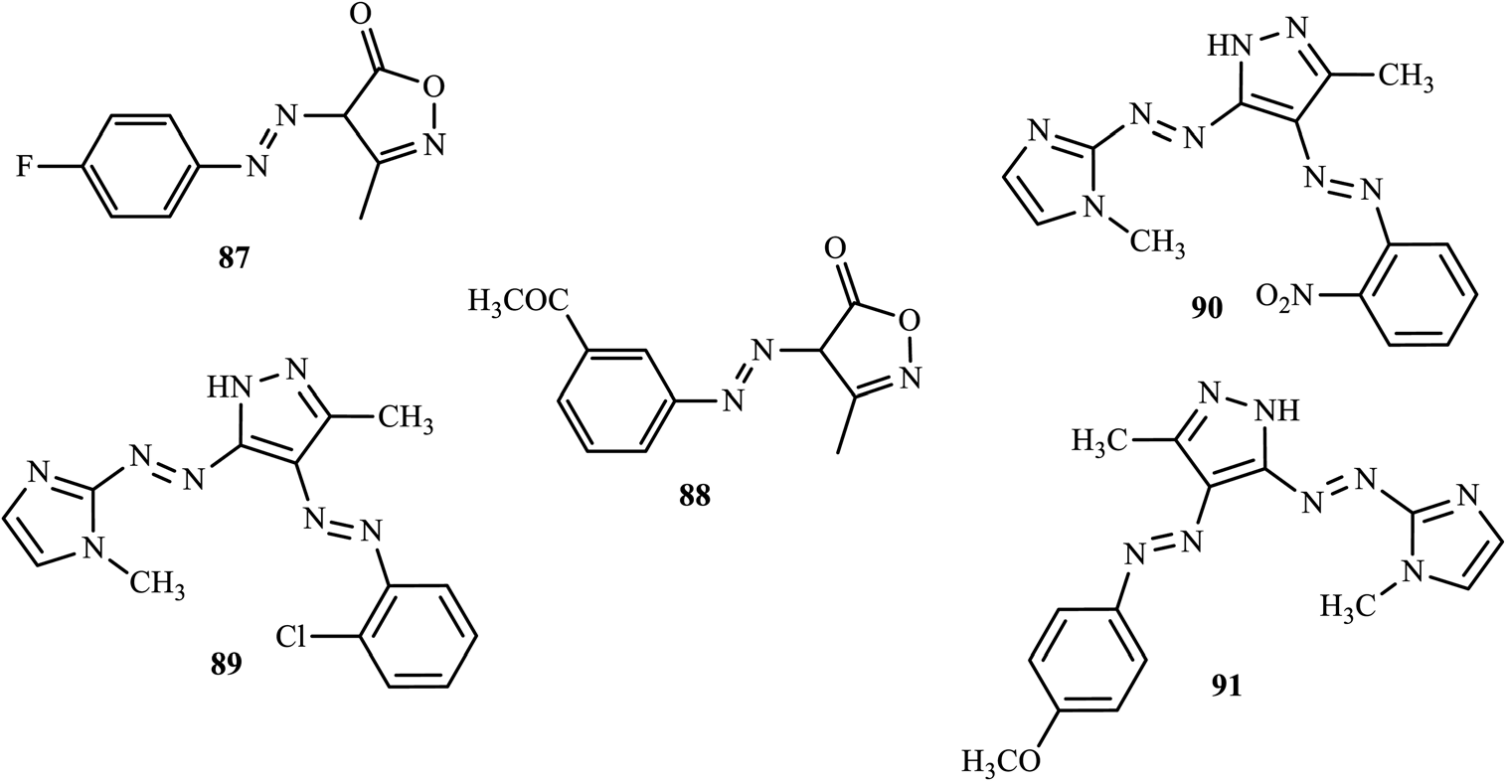
Chemical structures of antibacterial active azo dye derivatives incorporating heterocyclic moieties.

Banpurkar and co-workers reported the synthesis of 3-methyl-4*H*-isoxazol-5-one at room temperature by a simple stirring method from ethyl acetoacetate and hydroxylamine hydrochloride in an aqueous medium and coupled it with diazotized substituted amines to form a series of 4-(substituted phenylazo)-3-methyl-4*H*-isoxazol-5-ones through green chemistry.^41^ The antibacterial activities of the synthesized azo dyes were screened against *Escherichia coli*, *Pseudomonas aeruginosa*, *Staphylococcus aureus*, and *Staphylococcus pyogenus*. Both 4-(4-fluoro-phenylazo)-3-methyl-4*H*-isoxazol-5-one (87) and 4-(3-acetyl-phenylazo)-3-methyl-4*H*-isoxazol-5-one (88) (Fig. 3) showed good antibacterial activity against *Escherichia coli* and *Staphylococcus aureus*, close to that of ampicillin.

In a recent study, novel disazo dyes containing imidazole and pyrazole cycles were synthesized by Atay and co-workers, through diazotization-coupling.^42^ The antimicrobial activity of synthetic dyes was tested against a number of pathogenic bacteria (*Staphlococcus aureus* ATCC 25923, *Bacillus cereus* ATCC 10876, *Listeria monocytogenes* ATCC 7644) and the synthetic dyes 4-((2-chlorophenyl)diazenyl)-3-methyl-5-((1-methyl-1*H*-imidazol-2-yl)diazenyl)-1*H*-pyrazole (89), 3-methyl-5-((1-methyl-1*H*-imidazol-2-yl)diazenyl)-4-((2-nitrophenyl)diazenyl)-1*H*-pyrazole (90) and -methyl-5-((1-methyl-1*H*-imidazol-2-yl)diazenyl)-4-((4-methoxyphenyl)diazenyl)-1*H*-pyrazole (91) (Fig. 3) showed good antimicrobial activity.

### 3.2. Antifungal activity

**Fig. 4:**
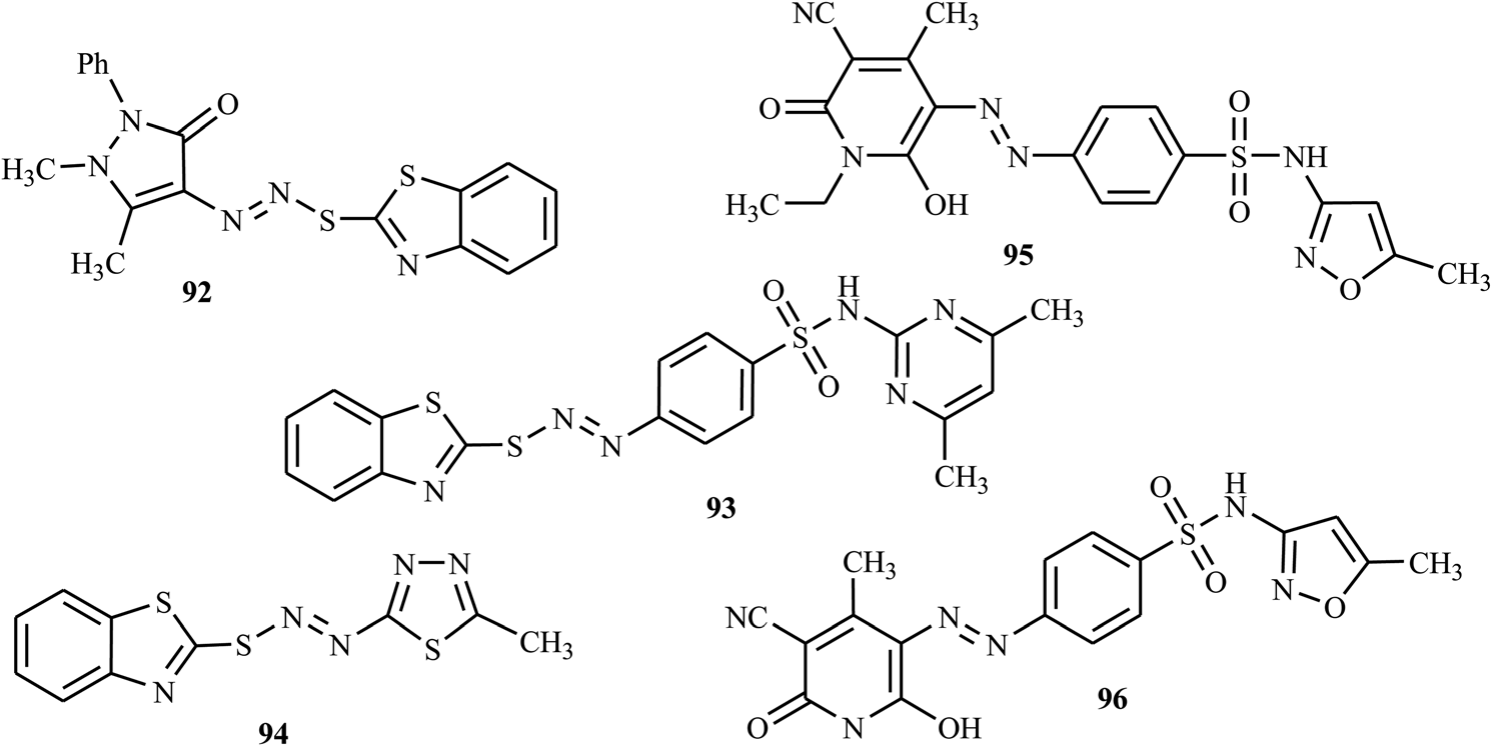
Chemical structures of antifungal active azo dye derivatives containing heterocyclic scaffolds.

Recently, Matada and colleagues reported new *S*-heterocyclic azo dyes synthesized from 1,3-benzothiazole-2-thiol with various amines by the diazo-coupling method.^43^ The azo molecules derived from benzothiazole were screened for their microbial inhibition by modified tube dilution assay against two fungal strains, *C. albicans*, and *A. flavus*, and the results were correlated with fluconazole. The antifungal activities of compounds 92, 93 and 94 (Fig. 4) showed promising results against *C. albicans* and *A. flavus*. The presence of heterocyclic rings in their structures contributed to the enhancement of antifungal activity, as described in ref. 44–46.

Mallikarjuna and Keshavayya synthesized and reported bright-colored heterocyclic azo dyes from sulfamethoxazole with various coupling compounds.^47^ The antifungal activity of these target compounds was studied against *A. flavus* and *C. albicans*, with the reference drug fluconazole, and synthesized azo dyes 95 and 96 (Fig. 4) were proven to have antifungal properties against two pathogenic strains, *viz. A. flavus* and *C. albicans*. Further, these azo dyes have shown promising antibacterial, anti-mycobacterial, and anticancer activity, which indicate that the compounds are efficient in inhibiting multiple diseases.

### 3.3. Anti-tuberculosis activity

**Fig. 5:**
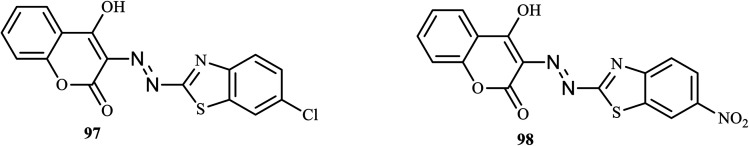
Chemical structures of antimycobacterial active azo dye derivatives containing the benzothiazole moiety.

In recent years, tuberculosis has become one of the most dangerous infectious diseases and a leading cause of death worldwide,^48^ and it is a challenge for researchers to design effective anti-TB drugs. An azo dye based on coumarin-benzothiazole was synthesized by Bodke and co-workers.^49^ The effectiveness of the synthesized dyes was tested against *Mycobacterium tuberculosis* (H37 RV strain) and compared to the standard drugs using the microplate Alamar Blue Assay method. Among the azo dyes, 3-(6-chloro-benzothiazol-2-ylazo)-4-hydroxy-chromen-2-one (97) and 4-hydroxy-3-(6-nitro-benzothiazol-2-ylazo)-chromen-2-one (98) (Fig. 5) exhibited similar excellent sensitivities (MIC = 1.6 μg mL^−1^) relative to the standard streptomycin (MIC = 6.24 μg mL^−1^).

### 3.4. Anticancer activity

**Fig. 6:**
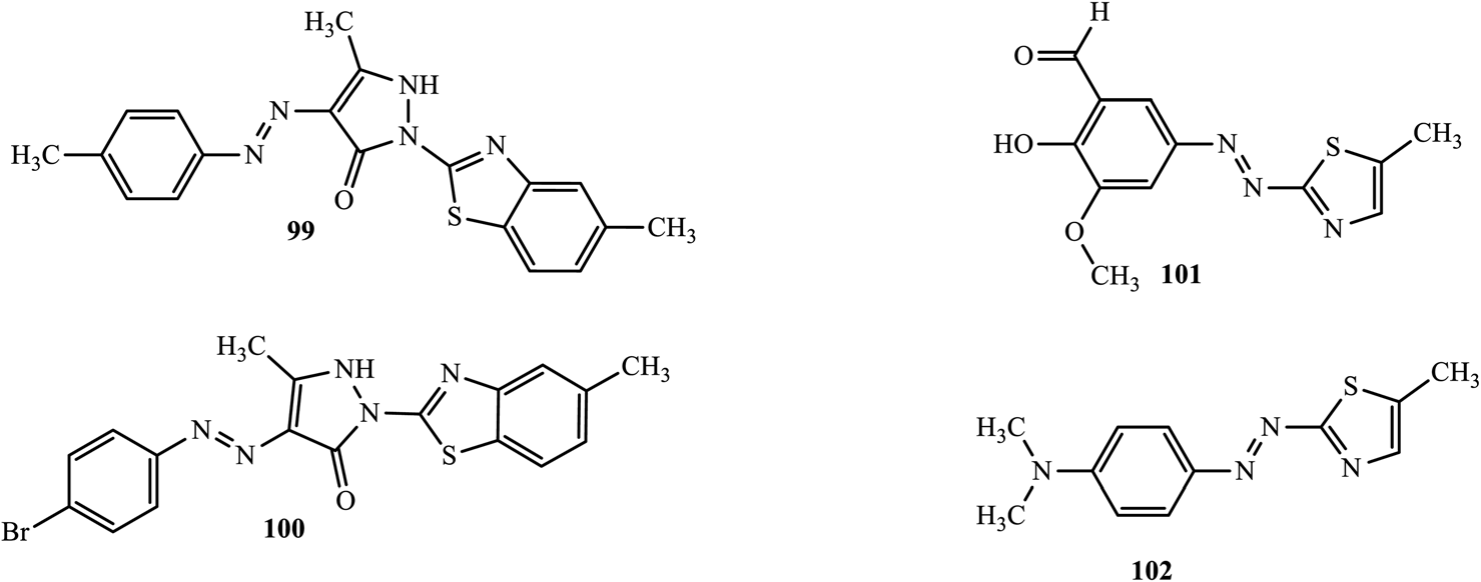
Chemical structures of anticancer active azo dye derivatives incorporating heterocyclic scaffolds.

Anticancer active novel heterocyclic azo dyes were synthesized and reported by Maliyappa and co-workers through a conventional diazo-coupling reaction.^50^ The anticancer activity of the compounds was studied against human cancer cell lines like the colon cell line (HCT116), lung carcinoma cell line (A549), T-lymphocyte cell line (Jurkat) and chronic myeloid leukemia cell line (K562). The 5-methyl-2-(5-methyl-benzothiazol-2-yl)-4-*p*-tolylazo-1,2-dihydro-pyrazol-3-one (99) and 4-(4-bromo-phenylazo)-5-methyl-2-(5-methyl-benzothiazol-2-yl)-1,2-dihydro-pyrazol-3-one (100) (Fig. 6) exhibited good activity towards the human colon cell line (HCT116) to inhibit the growth of the cancerous cells.

Keshavayya and colleagues also reported powerful anti-cancer active heterocyclic azo dyes 2-hydroxy-3-methoxy-5-(5-methyl-thiazol-2-ylazo)-benzaldehyde (101) and dimethyl-[4-(5-methyl-thiazol-2-ylazo)-phenyl]-amine (102), derived from 2-amino-5-methyl-thiazole by the diazo coupling reaction.^51^ The azo dyes were screened *in vitro* against A-549 and K-562 cell lines, and both compounds exhibited potent anticancer activity.

### 3.5. Anti-inflammatory activity

In a conventional diazo coupling reaction, Bodke and co-workers synthesized novel anti-mycobacterial isoxazolone-thiazole-based azo dyes,^7^ and evaluated the anti-inflammatory activity of the synthesized azo dyes against matrix metalloproteinase-2 (MMP-2) and matrix metalloproteinase-9 (MMP-9) using gelatin zymography. The azo dyes dimethyl-[4-(5-methyl-thiazol-2-ylazo)-phenyl]-amine (103) and 4-(benzothiazol-2-ylazo)-3-phenyl-2*H*-isoxazol-5-one (104) exhibited significant anti-inflammation activity against MMP-2 and MMP-9.

**Fig. 7:**
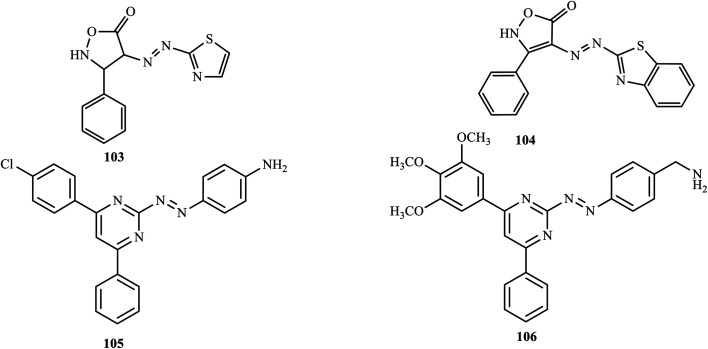
Chemical structures of anti-inflammatory active azo dye derivatives incorporating heterocyclic scaffolds.

In a recent paper, Unnisa and co-workers reported and synthesized pyrimidine azo dyes by coupling phenylpyrimidine 2-amine with different aromatic amines.^52^ The synthesized compounds were screened for their anti-inflammatory activities through the heat-induced hemolysis method. Most of the synthesized compounds exhibited a membrane stabilization effect by inhibiting the lysis of the erythrocyte membrane. Compounds 105 and 106 (Fig. 7) showed maximum inhibitory activities of 71.08%, and 71.91%, respectively, which are closer to the standard aspirin (72.91%).

### 3.6. Antioxidant activity

**Fig. 8:**
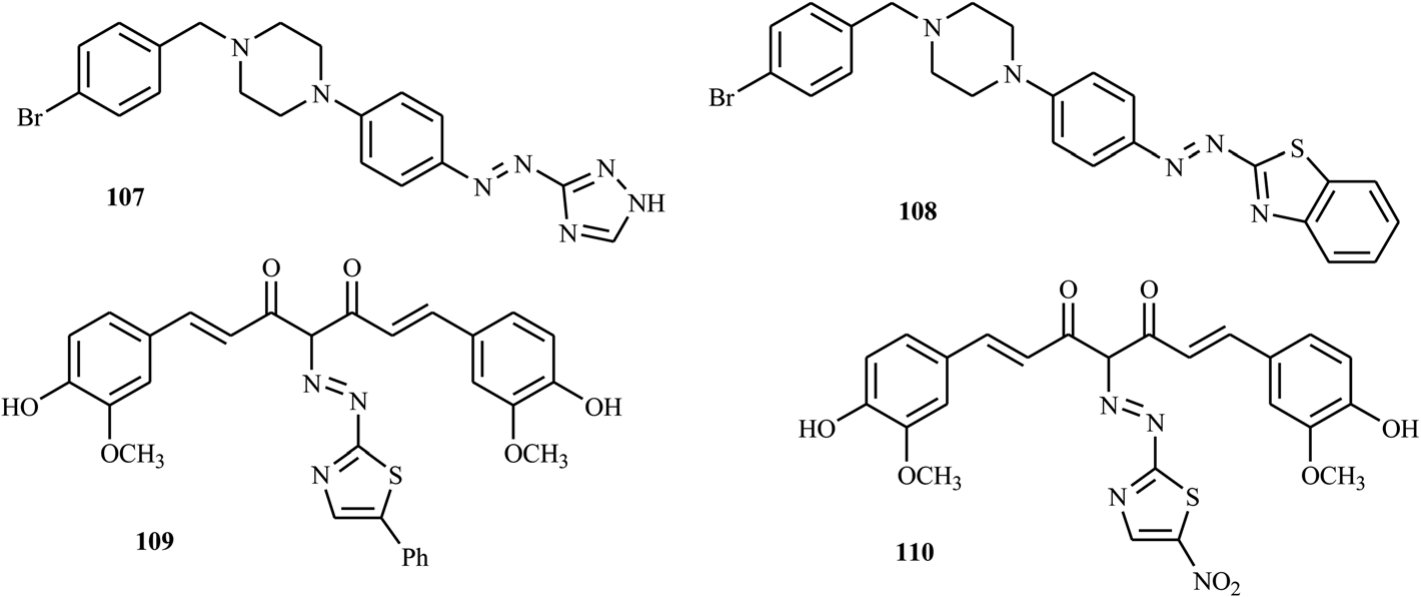
Chemical structures of antioxidant active heterocycle-incorporated azo dye derivatives.

A series of novel bioactive disperse dyes (Fig. 8) consisting of thiazolyl and piperazine moieties were reported by Mohammadi and co-workers *via* azo coupling reactions.^53^ The antioxidant activities of the newly synthesized compounds were evaluated by FRAP. All of the compounds displayed significant antioxidant activity. Among the tested dyes, azo dye 107 and 108 exhibit good radical scavenging activity. The activities of these compounds were attributed to the presence of thiazolyl derivatives and piperazine moieties as bioactive components in the structures of synthesized dyes.^53^

Abu-Melha and co-workers reported the synthesis of novel bioactive thiazolyl-curcumin azo dyes in which curcumin was coupled with different aromatic diazonium salts of 2-amino thiazole derivatives, such as 2-aminobenzothiazole, 2-amino-5-phenylthiazole, 2-amino-5-methylthiazole and 2-amino-5-nitrothiazole.^54^ All synthesized compounds were tested, and their antioxidant activities reflected the ability to inhibit oxidation. The antioxidant activities of the synthesized compounds were examined by ABTS inhibition, and compounds 109 and 110 showed higher antioxidant activity, comparable to ascorbic acid as a standard. Furthermore, the synthesized thiazolyl-curcumin derivatives exhibited promising antimicrobial, anticancer and antioxidant activities.

### 3.7. Antiviral activity

**Fig. 9:**
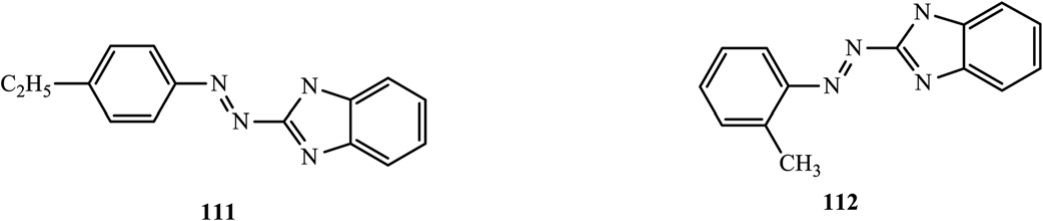
Chemical structures of antiviral active azo dye derivatives containing the benzoimidazole scaffold.

Yellow-colored heterocyclic azo dye derivatives (1*H*-benzoimidazol-2-yl)-(4-ethyl-phenyl)-diazene (111) and (1*H*-benzoimidazol-2-yl)-*o*-tolyl-diazene (112), which are antiviral in nature, were synthesized and reported by Mohammad Ashfaq.^55^ The compounds were tested *in vivo* against viruses in developing chick embryos. Labels were applied to nine-day-old embryonated chicken eggs based on the compound used. As a result of the hemagglutination test in the case of the anti-NDV potential of the compounds for 100% at 0.1 mg/100 μl, both compounds inhibited 50% of NDV and AIV (H9N2) viral growth (Fig. 9).

## 4. Conclusion

Azo dyes incorporating heterocyclic scaffolds generate the largest volume of dye production, and they are regularly used in the food, pharmaceutical, paper, cosmetics, textile, and leather industries, among others. Nowadays, researchers are exploring the biological activities of various azo dyes by incorporating heterocyclic components in the synthesis, and the resulting dyes have enhanced applications in a wide range of fields, especially pharmaceuticals. Previously, azo dyes were synthesized through diazotization but nowadays, researchers are synthesizing various azo dyes and incorporating heterocyclics through diazotization coupling reactions followed by post-modification methods, thus improving their biological and pharmaceutical activities. Due to the potential chemistry of azo dyes and their derivatives that incorporate heterocyclic scaffolds, there is much to contribute toward the discovery of new, potent and bioactive drugs with a broad spectrum of activities. Therefore, the synthesis of azo dyes with heterocyclic moieties requires further investigation to enhance the pharmacological activities, leading to the development of new drugs.

## Conflicts of interest

There are no conflicts to declare.

## Supplementary Material
